# Supplementary material for: Novel Approach for Evaluating Pregnancy-Associated Glycoprotein and Inflammation Markers during the Postpartum Period in Holstein Friesian Cows
Source: Animals (Basel). 2024 May 14;14(10):1459. doi: 10.3390/ani14101459 (PMC11117242; doi:10.3390/ani14101459)
Supplement: Supplementary file 1 [file animals-14-01459-s001.zip › animals-2978315-supplementary.pdf]

Table S1. Characteristics of the cows included in this study.

Parameters	No.
Cows examined	19
Analyzed cows	19
Postpartum status	
Pregnant	13 (68.4%)
First AI pregnancy rate	21.0%
Culled	2
Non-pregnant	4
Days open period	
Mean ± SD	121.5 ± 53.0
Median (range)	105 (68, 246)
No. of cows with cysts	12 (63.1%)
No. of cows with mastitis	11 (57.8%)
No. of cows with ketosis	8 (42.1%)
No. of cows with endometritis, metritis, or pyometra	4 (21.0%)

SD, standard deviation; AI, artificial insemination.

Table S2. Association between mastitis, PAG slope, postpartum days open period, and inflammation markers.

	Mastitis		*p*-Value
	Without	With	Without vs. with
No. of cows	8	11	
Days open period (mean ± SD)	119.5 ± 36.9	133.1 ± 67.8	0.640
PAG slope	−0.066 ± 0.018	−0.047 ± 0.028	0.095
Estimate day 1 SAA	2.44 ± 1.01	2.59 ± 1.08	0.762
SAA slope	−0.039 ± 0.005	−0.041 ± 0.006	0.706
No. of cows with MAA more than 12 μg/mL (%)	3 (37.5)	9 (81.8)	0.074

SD, standard deviation; PAG, pregnancy-associated glycoprotein; SAA, serum amyloid A; MAA, milk amyloid A.

Table S3. Association between ketosis, PAG slope, postpartum days open period, and inflammation markers.

	Ketosis		*p*-Value
	Without	With	With vs. without
No. of cows	11	8	
Days open period (mean ± SD)	116.0 ± 44.4	134.0 ± 75.3	0.679
PAG slope	−0.059 ± 0.029	−0.056 ± 0.010	0.810
Estimate day 1 SAA	2.62 ± 1.16	2.29 ± 0.95	0.514
SAA slope	−0.040 ± 0.006	−0.038 ± 0.008	0.653
No. of cows with MAA more than 12 μg/mL (%)	7 (63.6)	5 (62.5)	0.999

SD, standard deviation; PAG, pregnancy-associated glycoprotein; SAA, serum amyloid A; MAA, milk amyloid A.
